# Inadvertent Trypan Blue Staining of Posterior Capsule during Cataract Surgery Associated with “Argentinian Flag” Event

**DOI:** 10.1155/2016/9025063

**Published:** 2016-02-28

**Authors:** Robert A. Prinzi, Neeti M. Alapati, Shawn S. Gappy, Jason S. Dilly

**Affiliations:** ^1^Department of Ophthalmology, Henry Ford Hospital, 2799 West Grand Boulevard, Detroit, MI 48202, USA; ^2^Department of Ophthalmology and Visual Sciences, Washington University School of Medicine, 660 South Euclin Avenue, McMillan Building, Box 8096, Saint Louis, MO 63110, USA

## Abstract

Trypan blue is common in visualizing the anterior capsule during cataract surgery. Inadvertent staining of the posterior capsule during phacoemulsification is a rare complication and there are few reports in the literature. The proposed mechanism of posterior capsule staining in previous reports includes a compromised zonular apparatus or iris retractors facilitating the posterior flow of trypan blue. We report the first case of trypan blue staining of the posterior capsule associated with the “Argentinian flag” sign. In our case, the “Argentinian flag” allowed the trypan blue to seep between the posterior capsule and the lens, staining the anterior surface of the posterior capsule.

## 1. Introduction

Trypan blue is a dye compound commonly used in cataract surgery to help visualize the anterior capsule of dense cataracts and to facilitate the creation of a continuous curvilinear capsulorhexis [1]. It has also shown utility in staining the posterior capsule to assist with completing a posterior capsulorhexis in pediatric cataract surgery [2]. In practice, there have been few reports of inadvertent staining of the posterior capsule with trypan blue during cataract surgery. We report a rare case of trypan blue staining of the posterior capsule associated with the “Argentinian flag” sign.

## 2. Case Report

The patient is a 36-year-old African American male who presented with a chief complaint of severely limited vision in both eyes. He denied any history of previous ocular surgery, trauma, or systemic health problems. Slit lamp exam revealed dense white cataracts in both eyes with a best corrected visual acuity of hand motion in the right eye and 20/80 in the left eye. No phacodonesis or iris sphincter tears were present on examination. B-scan demonstrated the retina to be flat in both eyes. The patient first underwent uncomplicated phacoemulsification with intraocular lens implantation in the right eye facilitated by trypan blue capsule staining.

**Figure 1:**
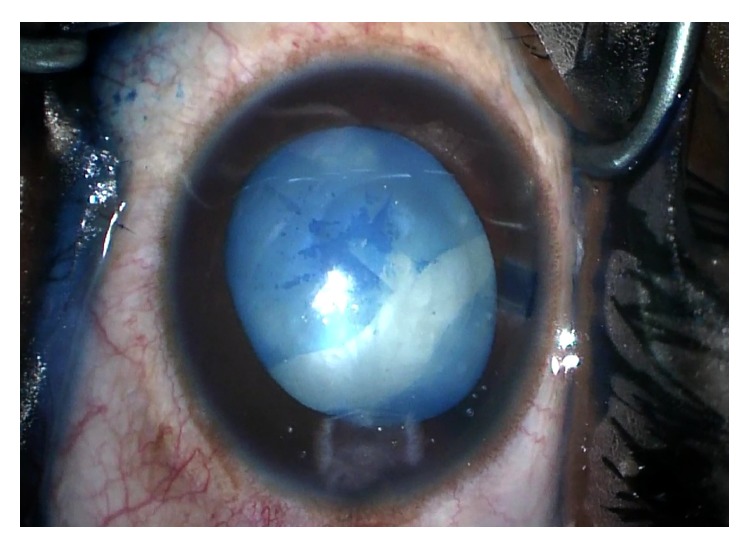
This photo demonstrates the “Argentinian flag” sign with the capsulorhexis extending peripherally.

**Figure 2:**
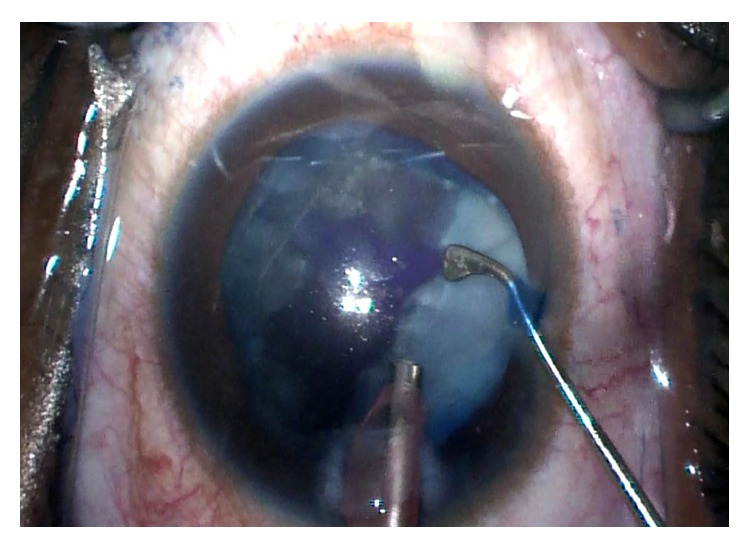
Trypan blue dye is noted on the posterior capsule during phacoemulsification.

**Figure 3:**
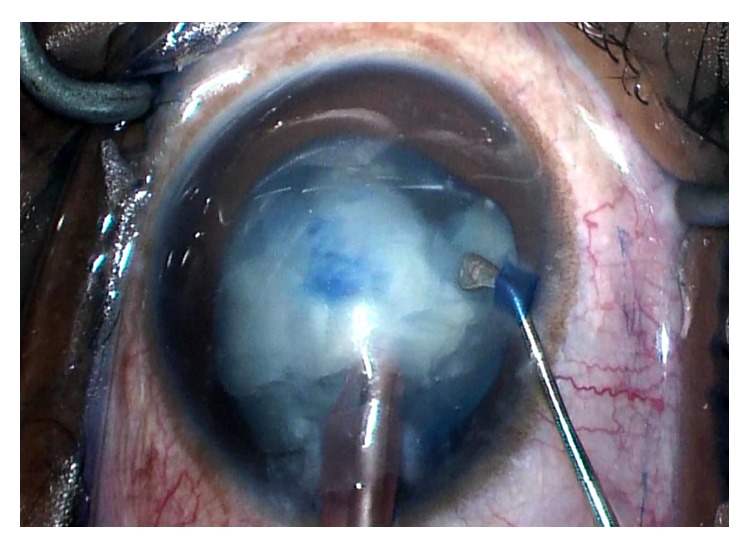
This photo demonstrates a lens fragment that flipped as it was being emulsified. Note the trypan blue staining on the formerly posterior surface of the lens fragment. This suggests that the Argentinian flag allowed access of the trypan blue to the anterior surface of the posterior capsule.

**Figure 4:**
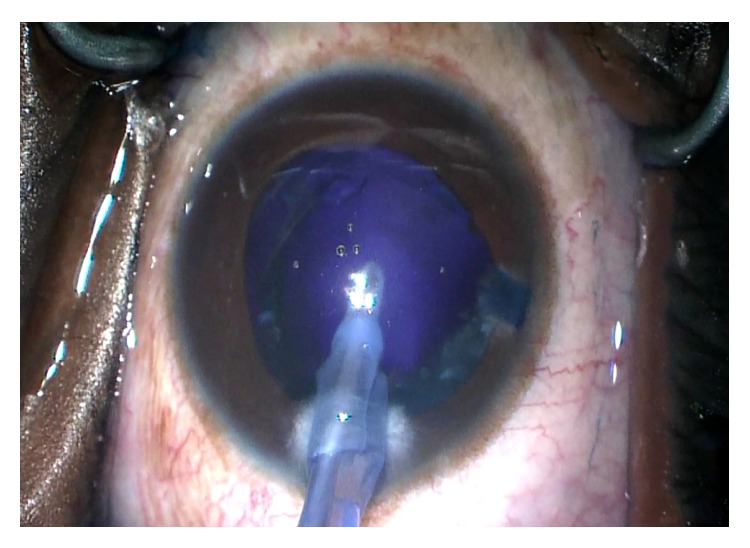
Photograph after cortex removal with irrigation and aspiration. Notice the absence of red reflex and the blue hue of the posterior capsule.

For the left eye, a similar procedure with the use of trypan blue and air bubble technique was planned. After the paracentesis was made, the anterior chamber was filled with an air bubble and the anterior capsule stained with trypan blue without incident. A dispersive ophthalmic viscoelastic device was injected into the anterior chamber replacing the air and a 2.4 mm triplanar clear-corneal incision was created. A small central tear created with the cystotome was promptly followed by the extrusion of some milky white nuclear material. The capsulorhexis began to extend peripherally, creating a partial “Argentinian flag” sign. Vannas scissors were used to begin a reverse capsulorhexis, but this incision also extended peripherally resulting in a full “Argentinian flag” sign (Figure 1). A can-opener technique was then performed, and once there was a safe flap to utilize, the capsulorhexis was continued and completed without incident. Gentle hydrodissection allowed the lens nucleus to prolapse into the anterior chamber. Nucleus and epinucleus were removed predominantly by aspiration with the Phaco Tip. During this step, the posterior capsule was noted to have a deep blue hue (Figure 2). Blue dye was also noted on the posterior surface of a lens fragment as it was aspirated (Figure 3). The remaining cortex was removed with irrigation and aspiration without incident (Figure 4). Lens removal was not hampered due to the blue staining given the density of the lens, which allowed it to be divided and conquered nicely with minimal cortical removal. The capsular bag was inflated using Provisc and an SN60WF one-piece intraocular lens (Alcon Laboratories, Inc., Fort Worth, Texas) was inserted into the capsular bag. It was checked to ensure that it was central and stable.

On postoperative day 1, the patient complained that his vision of his newly operated left eye was blue-tinged. Vision from that eye had improved to 20/70. There was no evidence of wound leak or residual lens material. Postoperative instructions were given and he was asked to return in 1 week. Unfortunately, he did not follow up as requested and has failed to return to the clinic despite insistence from his surgeon.

## 3. Discussion

Inadvertent staining of the posterior capsule with trypan blue is an uncommon occurrence. To our knowledge, there have been seven individual case reports and one small case series reporting unintentional staining of the posterior lens capsule during cataract surgery. Of the case reports, three reported a history of ocular trauma [3–5], but no clinical signs of zonular weakness were noted. One event occurred in a patient with pseudoexfoliation syndrome [6]. Another occurred in a patient with a previous vitrectomy for a rhegmatogenous retinal detachment [7]. In these cases, the proposed mechanism of posterior capsule staining was leakage of the trypan blue through a compromised zonular apparatus.

We found two case reports in which the posterior capsule was stained with trypan blue without a history of trauma or previous ocular surgery. Marques et al. reported posterior capsule staining during an extracapsular cataract extraction [8]. Pelit reported a case of a white cataract in a 45-year-old female with no history of trauma or prior eye surgery [9]. Of note, the female in that case had bilateral dense white cataracts. In both reports, the authors proposed that the posterior capsule staining was secondary to a high pressure dispersal of trypan blue, forcing the dye posteriorly through intact zonules.

In the case series mentioned above, Burkholder et al. reported five patients, all of whom required the use of iris retractors either to enlarge the pupil or to stabilize a floppy iris [10]. Only one of these patients reported a possible history of childhood trauma. The authors suggest that the iris retractors elevate the iris facilitating the posterior flow of trypan blue. They also postulate that intraoperative floppy iris syndrome may result in a greater amount of iris elevation and thus be an additional risk factor.

In our patient, we believe that the trypan blue seeped between the posterior capsule and the lens. The “Argentinian flag” allowed access of the dye to the anterior surface of the posterior capsule. Although it is difficult to be certain, the authors believe that only the posterior capsule was stained and that the vitreous remained unstained. The “Argentinian flag” did not extend past the equator or involve the posterior capsule. Therefore, we do not believe that the trypan blue had direct access to the vitreous. As shown in Figure 3, the posterior portion of the lens fragment was stained blue. It is possible that our patient had a compromised zonular apparatus, although there were no clinical signs to suggest this preoperatively or intraoperatively.

In previous reports, the posterior capsule staining resolves after approximately one week. Unfortunately, the patient did not return for further follow-up despite multiple requests from our office. He did not, however, report further blue-tinge to his vision afterward.

In conclusion, this case represents a rare instance of inadvertent posterior capsule staining with trypan blue, likely induced by the development of the “Argentinian flag” sign. We believe the “Argentinian flag” allowed the trypan blue access to the anterior surface of the posterior capsule. Although infrequently seen, it remains incumbent upon anterior segment surgeons to remain vigilant for such issues as the use of trypan blue comes into more widespread use.
